# Synthetic zipper mediated pre-targeting system for near-infrared photoimmunotherapy

**DOI:** 10.1016/j.isci.2025.114558

**Published:** 2025-12-29

**Authors:** T.M. Mohiuddin, Chaoyu Zhang, Wenjie Sheng, Marwah Al-Rawe, Natalia El-Merhie, Felix Zeppernick, Ivo Meinhold-Heerlein, Ahmad Fawzi Hussain

**Affiliations:** 1 Department of Gynecology and Obstetrics, Medical Faculty, Justus-Liebig-University Giessen, Klinikstr. 33, 35392 Giessen, Germany; 2 Department of Gynaecology and Obstetrics, Brandenburg Medical School Theodor Fontane, University Clinic Brandenburg, Hochstraße 29, 14770 Brandenburg an der Havel, Germany; 3 Institute for Lung Health (ILH), Cardiopulmonary Institute (CPI), Member of the German Center for Lung Research (DZL), Justus Liebig University Giessen, Aulweg 128, 35392 Giessen, Germany

**Keywords:** drug delivery system, conjugate, biological sciences, cell biology, cancer

## Abstract

Several strategies have gained attraction in cancer diagnosis and therapy, particularly to overcome the limitations associated with direct targeting approaches in radioimmunotherapy and photoimmunotherapy. Here, we have introduced a synthetic zipper-mediated pre-targeting system for near-infrared photoimmunotherapy (NIR-PIT) utilizing the IR700-conjugated Zip1-SNAP protein in combination with three scFv-ZIP2 fusion proteins targeting FOLR1, TROP2, and TF. In this two-step targeting approach, scFv-Zip2 proteins first bind to cancer-specific antigens, followed by hybridization with Zip1-SNAP-IR700 at cell surface via high-affinity zipper interaction. Upon NIR light irradiation, targeted cancer cells underwent selective phototoxicity in a concentration-dependent manner with IC_50_ range (∼104–1,179 nM). Notably, the treatment induced cell death in ∼86%–94% of cells and triggered ICD, as evidenced by increased (∼4– to 200-fold more) surface exposure of calreticulin, HSP70 and HSP90. These findings demonstrate that the synthetic zipper-mediated pre-targeting platform offers a promising and versatile strategy for enhancing the specificity and immunogenic potential of NIR-PIT in cancer therapy.

## Highlights

• Synthetic zipper enables pre-targeting of NIR-PIT in cancer cells
• ScFv-Zip2 fusion proteins target FOLR1, TROP2, and TF antigens
• Synthetic zipper mediated NIR-PIT systems induces potent cancer cell death
• Triggers immunogenic cell death via calreticulin and HSP exposure

## Graphical abstract

**Figure:**
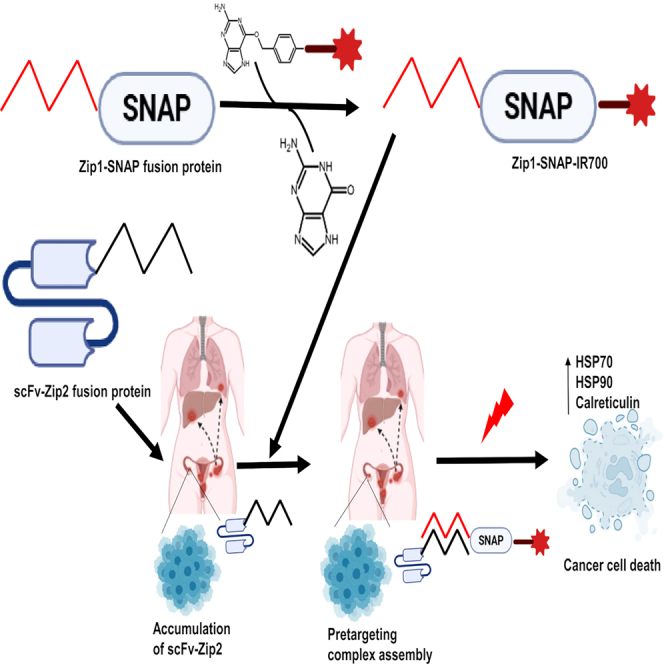

## Introduction

Pre-targeting is an emerging and promising strategy in cancer therapy designed to overcome the limitations of direct targeting by decoupling tumor localization from therapeutic activation. This two-step approach has shown particular utility in applications—such as radioimmunotherapy,^1^^,^^2^ photoimmunotherapy,^3^ and imaging.^4^^,^^5^ In a typical pre-targeting protocol, a tumor-specific targeting moiety—such as an antibody or ligand conjugated with a bioorthogonal reactive group—is first administered and allowed to localize to the tumor. Subsequently, a therapeutic payload (e.g., a radionuclide^6^ chemotherapeutic agent^7^ or photoactivatable dye^8^ bearing a complementary reactive group) is introduced. This payload binds selectively to the tumor-localized targeting agent, including cytotoxicity while minimizing systemic toxicity and enhancing therapeutic precision.^9^^,^^10^^,^^11^ Several biochemical strategies have been explored to engineer pre-targeting systems, including biorthogonal click chemistry,^7^^,^^12^ bispecific antibody/hapten systems,^12^ streptavidin/biotin interaction,^13^ and hybridization of complementary oligonucleotides.^14^^,^^15^

Targeted photoimmunotherapy (PIT) has emerged as a powerful cancer treatment strategy that combines the molecular specificity of immunotherapy with the spatiotemporal control of phototherapy. Recent developments have significantly advanced this field. For example, a highly efficient and selective PIT approach utilizing PD-L1 as a therapeutic target demonstrated remarkable tumor suppression and immune modulation.^16^ In parallel, near-infrared PIT (NIR-PIT) is a targeted cancer treatment modality that combines a monoclonal antibody (mAb) with the near-infrared photosensitizer IRDye700DX (IR700).^17^ Upon NIR light exposure, the IR700-conjugated mAb bound to the tumor surface causes rapid cell membrane damage, leading to cell swelling, blebbing, and necrotic death.^18^^,^^19^^,^^20^ NIR-PIT has shown therapeutic efficacy against several cancer types by targeting antigens such as epidermal growth factor receptor1 (EGFR), human epidermal growth factor receptor 2 (Her2), carcinoembryonic antigen (CEA), and prostate-specific membrane antigen (PSMA).^20^ In ovarian cancer, folate receptor α (FOLR1),^21^ trophoblast cell surface antigen 2 (TROP2),^22^^,^^23^^,^^24^^,^^25^ and tissue factor (TF)^26^^,^^27^ are frequently overexpressed and play key roles in tumor progression, making them attractive targets for therapy.

However, conventional NIR-PIT approaches involve direct chemical conjugation of IR700 to full-length mAbs, often resulting in heterogeneous products with variable activity.^17^^,^^28^ SNAP-tag technology provides a solution by enabling site-specific and stoichiometrically controlled conjugation of IR700-generating homogenous antibody conjugate through binding with benzylgunaine (BG)-modified IR700.^29^^,^^30^^,^^31^ Moreover, replacing full mAbs with single-chain variable fragments (scFvs) can improve tumor penetration and reduce systemic circulation time, enhancing the therapeutic window.^32^^,^^33^^,^^34^ Despite these advantages, most current NIR-PIT systems remain limited by their single-target design,^18^^,^^19^^,^^20^ requiring the development of individual mAb-IR700 conjugates for each target antigen—a process that is labor-intensive, costly, and not easily generalizable.

To address these limitations, we propose a synthetic zipper-based pre-targeting platform for NIR-PIT. Synthetic zippers are engineered coiled-coil peptides that form highly specific, orthogonal heterodimers and have been characterized as versatile molecular connectors in synthetic biology.^35^ In our system, tumor-specific scFv-Zip2 fusion proteins are used to target cancer-associated antigens (FOLR1, TROP2, and TF). After tumor localization, a complementary Zip1-SNAP fusion protein conjugated to IR700 is administered. The high-affinity interaction between Zip1 and Zip2 enables selective binding at the tumor site.^36^ Subsequent NIR light exposure induces targeted cytotoxicity and immunogenic cell death (ICD), as evidenced by necrosis and exposure of damage-associated molecular patterns (DAMPs) such as calreticulin, HSP70, and HSP90.

This study demonstrates that the synthetic zipper-mediated pre-targeting system offers a robust and modular alternative to conventional NIR-PIT approaches. It enables precise, antigen-agonistic delivery of therapeutic agents without the need for repeated conjugate synthesis, thereby expanding the versatility, efficiency, and translational potential of NIR-PIT for a wide range of cancers.

## Results

### Generation of synthetic zipper-mediated pre-targeting complex

**Figure 1:**
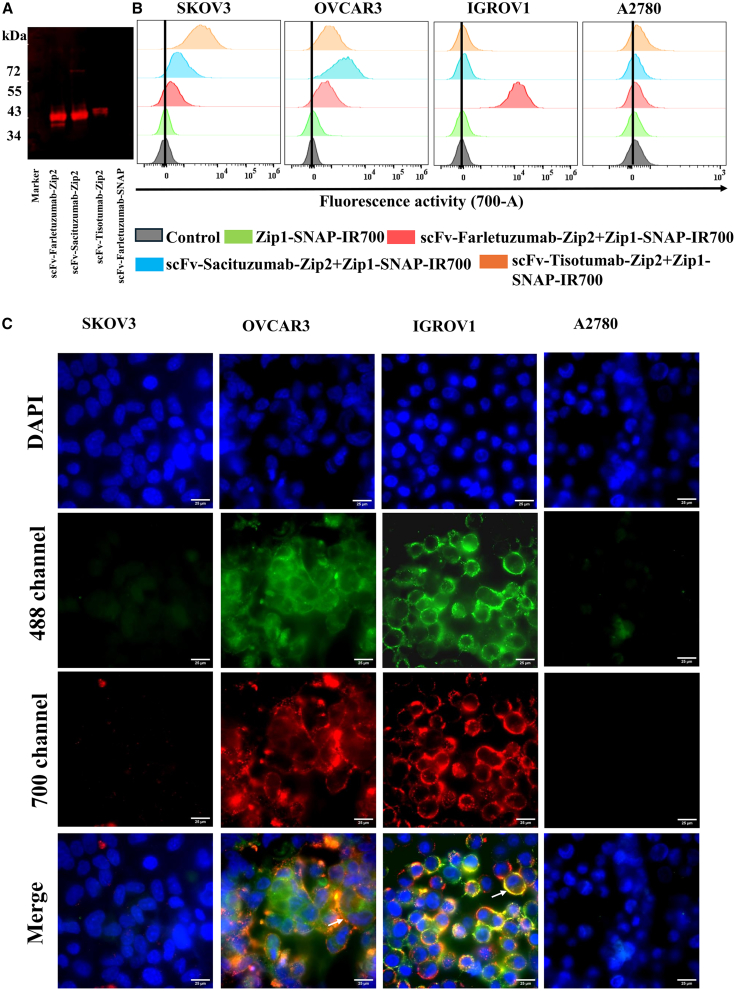
Specific interaction of scFv-Zip2 and Zip1-SNAP and their binding specificity in ovarian cancer cells (A) IR700 fluorescence was visualized using an Odyssey DLx Imager after incubation of scFv-Zip2 fusion proteins (scFv-Farletuzumab-Zip2, scFv-Sacituzumab-Zip2, and scFv-Tisotumab-SNAP-Zip2) with Zip1-SNAP-IR700. The scFv-Farletuzumab-SNAP was used as a control to assess the specificity of synthetic zipper interactions. (B) Flow cytometry analysis confirmed the antigen-specific binding of the pre-targeting complex in ovarian cancer cell lines. Histograms show the binding of scFv-Farletuzumab-Zip2 + Zip1-SNAP-IR700, scFv-Sacituzumab-Zip2 + Zip1-SNAP-IR700 and scFv-Tisotumab-Zip2 + Zip1-SNAP-IR700 to FOLR1^+^, TROP2^+^, and TF^+^ ovarian cancer cells. (C) scFv-Zip2 and Zip1-SNAP were conjugated with 488 and IR700 respectively. We showed only IR700 channel to visualize only the Zip1-SNAP-IR700 florescence signal and finally merged to 488 and DAPI to visualize the internalization. Fluorescence microscopy images showing co-localization of scFv-Farletuzumab-Zip2 labeled with Alexa Fluor 488 and Zip1-SNAP-IR700 on ovarian cancer cells at 4°C, confirming the specific cell surface interaction of the pre-targeting complex. Scale bars, 25 μm.

To develop synthetic zipper-mediated pre-targeting complex, scFv-Zip2 and Zip1-SNAP fusion proteins were successfully generated by fusing respective coding sequence and expressing them in expression vector (Figure S1). The Zip1-SNAP fusion protein was conjugated with BG-IR700 following the protocol described by Zhang et al.^37^ The specific interaction between scFv-Zip2 and Zip1-SNAP was confirmed by fluorescence western blot analysis. The scFv constructs—scFv-Farletuzumab-Zip2, scFv-Sacituzumab-Zip2, and scFv-Tisotumab-Zip2 were visualized using IR700 conjugated Zip1-SNAP in PVDF membrane and imaged at 700 nm with an Odyssey DLx Imager (LI-COR Bioscience) (Figure 1A). These results confirmed the successful interaction between the scFv-Zip2 and Zip1-SNAP fusion proteins.

### FOLR1-, TROP2-, and TF-targeted pre-targeting complex of NIR-PIT selectively bind to ovarian cancer cells

**Figure 2:**
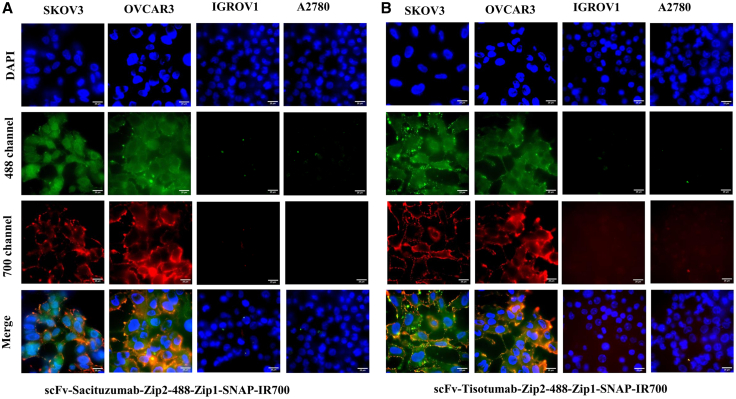
Specific binding and co-localization of pre-targeting complex (A) Cells were incubated with Alexa Fluor 488-labeled (A) scFv-Sacituzumab-Zip2 and (B) scFv-Tisotumab-Zip2, followed by treatment with Zip1-SNAP-IR700. Fluorescence microscopy at 4°C revealed specific binding and co-localization of the scFv-Zip2 constructs with Zip1-SNAP-IR700, confirming zipper-mediated interaction and spatial overlap of the pre-targeting components. Scale bars, 25 μm.

Flow cytometric analysis showed differential expression of FOLR1, TROP2, and TF in ovarian cancer cells (Figure S2). To assess the binding specificities of the scFv-SNAP tag proteins, SNAP-Surface Alexa Fluor 647 was conjugated to the fusion proteins and analyzed by flow cytometry. The results revealed binding to ovarian cancer cells that was dependent on the expression pattern of the target proteins (Figure S3). To assess the binding specificity of FOLR1-, TROP2-, and TF-targeted pre-targeting complex for NIR-PIT, ovarian cancer cell lines (SKOV3, OVCAR3, IGROV1, and A2780) were incubated with scFv-Farletuzumab-Zip2, scFv-Sacituzumab-Zip2, and scFv-Tisotumab-Zip2, followed by incubation with Zip1-SNAP-IR700, and analyzed via flow cytometry. The binding and co-localization of the scFv-Zip2 and Zip1-SNAP were further validated through fluorescent microscopy using the Zip1-IR700 and Alexa Fluor 488 conjugated scFv-Zip2. Flow cytometry and fluorescence imaging demonstrated that scFv-Farletuzumab-Zip2 specifically bound to the FOLR1-high-expressing OVCAR3 and IGROV1 cells, while only moderate to minimal fluorescence shifts were observed in the FOLR1-moderate and -low expressing SKOV3 and A2780 cells, respectively (Figure 1B). Co-localization of scFv-Zip2 and Zip1-SNAP was evident on the membrane of OVCAR3 and IGROV1 cells, as shown by overlapping signals from scFv-Farletuzumab-Zip2-488 and Zip1-SNAP-IR700 (Figure 1C). Similarly, strong binding signal and membrane co-localization were observed in SKOV3 and OVCAR3 cells with high expression of TROP2 and TF, respectively, when treated with scFv-Sacituzumab-Zip2+ Zip1-SNAP and scFv-Tisotumab-Zip2+ Zip1-SNAP, (Figures 2A and 2B).

### Pre-targeting complex of NIR-PIT selectively depletes FOLR1-, TROP2-, and TF-expressing ovarian cancer cells

**Figure 3:**
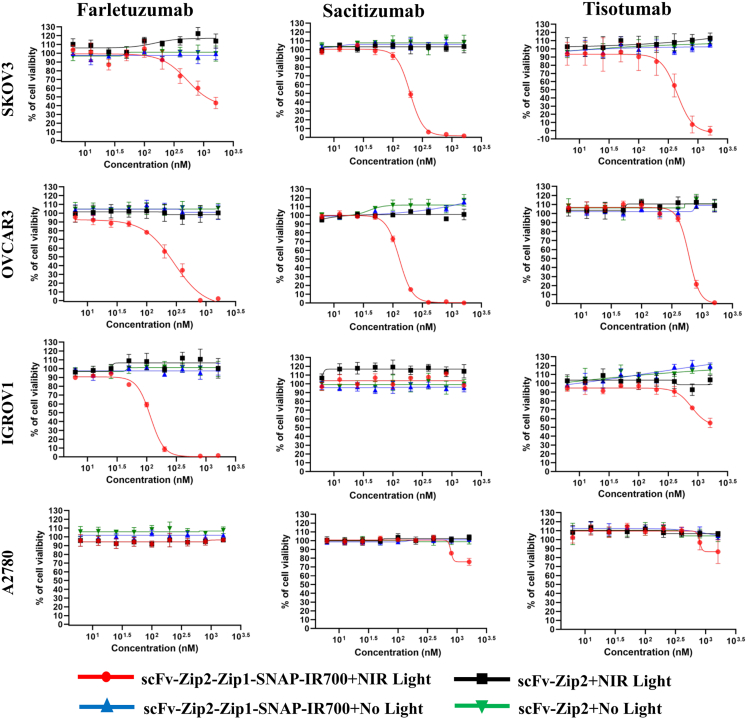
Antigen-specific cytotoxicity induced by the synthetic zipper-mediated pre-targeting complex in ovarian cancer cells Ovarian cancer cell lines were treated with increasing concentration (12.5, 25, 50, 100, 200, 400, 800, and 1,600 nM) of scFv-Zip2 fusion proteins targeting FOLR1 (scFv-Farletuzumab-Zip2), TROP2 (scFv-Sacituzumab-Zip2), and TF (scFv-Tisotumab-Zip2), followed by incubation with Zip1-SNAP-IR700. Cells were then exposed or not exposed to NIR light (690–710 nm LED, 2 J/cm^2^) and incubated at 37°C for 24 h. Cell viability was assessed using the XTT Cell Proliferation Kit II. Data represent mean ± SD from six biological replicates.

To evaluate the cytotoxic efficacy of the synthetic zipper-mediated pre-targeting system in NIR-PIT, a panel of ovarian cancer cell lines was treated with scFv-Zip2 fusion proteins scFv-Farletuzumab-Zip2, scFv-Sacituzumab-Zip2, and scFv-Tisotumab-Zip2. Following incubation with Zip1-SNAP-IR700, cells were exposed to 690–710 nm red light-emitting diode light at 2 J/cm^2^. Cell viability was assessed 24 h post-irradiation. Treatment with the scFv-farletuzumab-Zip2 + Zip1-SNAP-IR700 complex resulted in dose-dependent cytotoxicity in FOLR1-expressing SKOV3, OVCAR3, and IGROV1 cells, with IC_50_ values of 1,179 nM, 221.5 nM and 104.5 nM, respectively (Table 1). The scFv-Sacituzumab-Zip2 + Zip1-SNAP-IR700 complex similarly induced significant cytotoxicity to TROP2-positive SKOV3 and OVCAR3 cells, with IC_50_ values of 197.4 nM and 129.1 nM, respectively (Figure 3). Likewise, TF-targeted NIR-PIT using scFv-Tisotumab-Zip2 + Zip1-SNAP-IR700 demonstrated selective cytotoxicity in SKOV3 and OVCAR3 cells, which overexpress TF, with IC_50_ values 403.8 nM and 642.9 nM, respectively. A modest cytotoxic effect was observed in IGROV1 cells at the highest doses tested. In contrast, A2780 cells, which lack significant expression of FOLR1 and TF, showed no detectable cytotoxicity upon treatment with any of the three pre-targeting complexes, confirming the antigen-specific nature of the synthetic zipper-mediated NIR-PIT strategy. In addition, Zip1-SNAP-IR700 with or without light treatment showed no effect in SKOV3 cells. Moreover, the IC_50_ is negatively correlated (r = −0.86, *p* = 0.0119) with the expression level of target antigen (Figure S4B).

**Table 1:** IC_50_ values (nM) of the synthetic zipper-mediated pre-targeting complex of NIR-PIT in ovarian cancer cell lines

Target antigen	Pre-targeting complex of NIR-PIT	SKOV3	OVCAR3	IGROV1	A2780
FOLR1	scFv-Farletuzumab-Zip2 + Zip1-SNAP-IR700	1,179 ± 190.8	221.5 ± 22.2	104.5 ± 6.6	>1,600
TROP2	scFv-Sacituzumab-Zip2 + Zip1-SNAP-IR700	197.4 ± 11.6	129.1 ± 2.7	>1,600	>1,600
TF	scFv-Tisotumab-Zip2 + Zip1-SNAP-IR700	403.8 ± 57.4	642.9 ± 53.2	>1,600	>1,600

>1,600 indicates no significant cytotoxicity observed at the highest tested concentration.

### Induction of cell death in ovarian cancer cells by the pre-targeting complex of NIR-PIT

**Figure 4:**
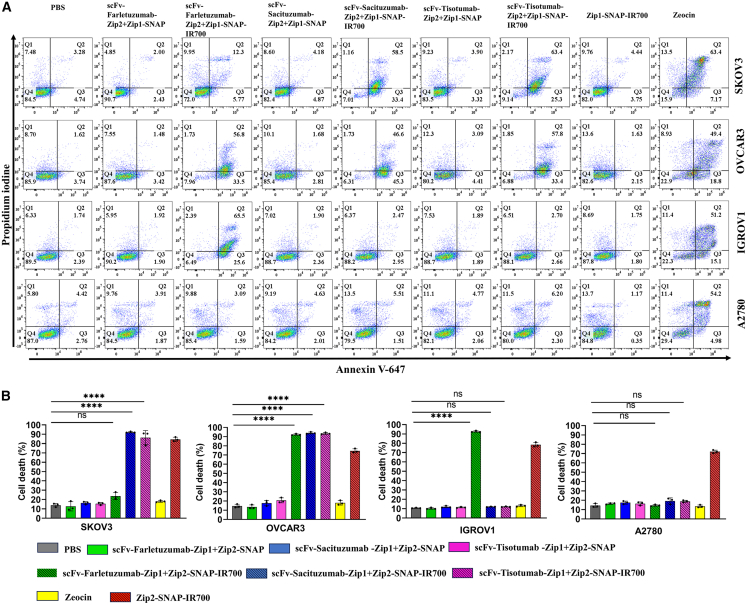
Induction of cell death in ovarian cancer cells following treatment with the pre-targeting complex for NIR-PIT (A) Cell death was assessed using annexin V assay 24 h post-treatment. Flow cytometric data are displayed as scatterplots. (B) Quantitation of cell death is shown as bar graphs. Data are represented as mean ± SD from three biological replicates. Cells incubated with media or zeocin were used as negative or positive control. The statistical analysis was carried out using Welch one-way ANOVA and Dunnett's test. ∗∗∗∗*p* ≤ 0.0001.

To determine whether the pre-targeting complexes targeting FOLR1, TROP2 and TF induce cell death following NIT-PIT, we performed annexin V/propidium iodide (PI) staining 24 h after light irradiation, following the protocol by Zhang et al.^37^ Flow cytometry analysis revealed that treatment with scFv-Farletuzumab-Zip2 + Zip1-SNAP-IR700, scFv-Sacituzumab-Zip2 + Zip1-SNAP-IR700, scFv-Tisotumab-Zip2 + Zip1-SNAP-IR700 resulted in significant cell death in SKOV3 cells. The majority of dying cells were annexin V^+^/PI^+^, indicative of late apoptosis/necroptosis, with a minor population undergoing early apoptosis (annexin V^+^/PI^−^). In OVCAR3, all three pre-targeting complexes induced substantial cell death compared to untreated controls, again predominantly through late apoptosis/necroptosis. Notably, only the Farletuzumab-Zip2 + Zip1-SNAP-IR700 triggered significant cell death in IGROV1 cells, consistent with their antigen expression profile. In contrast, A2780 cells showed no significant increase in cell death across any treatment group, underscoring the antigen specificity of the pre-targeting NIR-PIT system (Figure 4).

### Release of DAMPs from dying ovarian cancer cell treated with the pre-targeting complex of NIR-PIT

**Figure 5:**
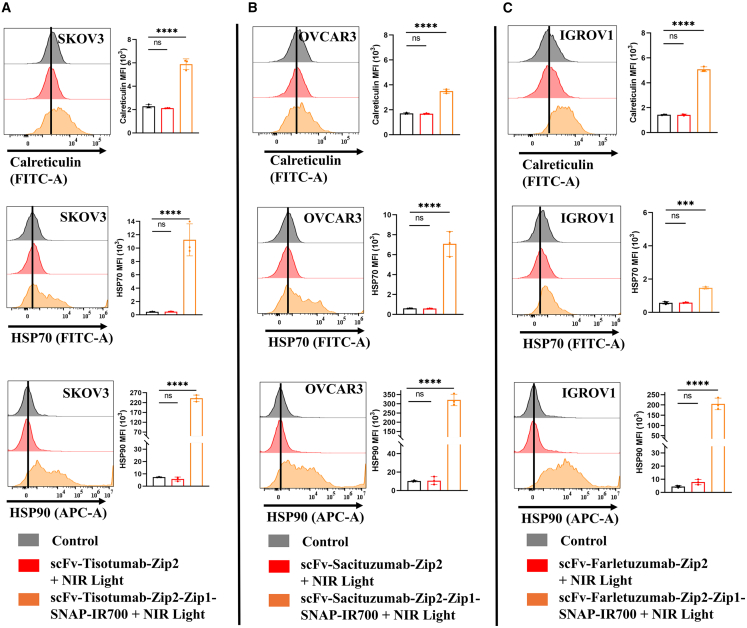
The pre-targeting complex of NIR-PIT induces the release of ICD markers (A) Flow cytometric histograms show cell surface expression of calreticulin, HSP70, and HSP90 compared to untreated controls in SKOV3 cells with scFv-tisotumab-Zip2-Zip1-SNAP-IR700. (B) OVCAR3 cells treated with scFv-Sacituzumab-Zip2-Zip1-SNAP-IR700. (C) IGROV1 cells treated with scFv-Farletuzumab-Zip2-Zip1-SNAP-IR700. Only viable cells were included in the analysis. Bar graphs show the mean fluorescence intensity (MFI) of cell surface calreticulin, HSP70, and HSP90. Data are represented as mean ± SD from three biological replicates. Cells exposed to NIR light irradiation without incubation with NIR-PIT agents were used as control. Statistical significance was determined by a one-way ANOVA and Dunnett's test. ∗∗∗*p* ≤ 0.001, ∗∗∗∗*p* ≤ 0.0001.

To investigate the immunogenic potential of NIR-PIT, we examined the release of DAMPs, which are key markers of ICD following therapy. IGROV1 cells treated with the FOLR1-targeted pre-targeting complex exhibited a significant upregulation of calreticulin on the cell surface. Flow cytometry analysis confirmed a substantial increase in surface calreticulin expression compared to untreated controls. In addition, increased expression levels of HSP70 and HSP90 were observed, further validating the induction of ICD. Similarly, OVCAR3 cells treated with the TROP2-targeted pre-targeting complex showed marked upregulation of calreticulin, HSP70, and HSP90. Likewise, treatment of OVCAR3 and SKOV3 cells with the TF-targeted NIR-PIT complex led to elevated expression of these DAMPs compared to untreated controls. These findings underscore the dual functionality of NIR-PIT agents in mediating targeted cytotoxicity while promoting immune activation through the release of immunogenic DAMP (Figure 5).

## Discussion

Cancer cells within tumors often exhibit heterogeneous expression of surface antigen, which limits the effectiveness of conventional antibody-based immunotherapies with fixed target specificities.^38^ Developing IR700-mAb conjugate for each antigen is impractical for clinical use. Pre-targeting strategies have been explored in preclinical radioimmunotherapy studies to address this limitation. These approaches decouple the targeting moiety from the therapeutic agent (e.g., radionuclide), allowing rapid clearance of unbound small molecules from circulation and enabling the use of short-lived radionuclides with reduced off-target toxicity.^2^^,^^39^^,^^40^^,^^41^

A recent study described a pre-targeting system for PIT utilizing IR700-conjugated NeutrAvidin and various biotinylated antibodies. This AvIR-based system demonstrated target-specific cancer cell killing.^3^ However, the use of avidin may pose immunogenicity concerns in clinical settings. To overcome this limitation, we developed a synthetic zipper mediated pre-targeting system that employs scFv-Zip2 fusion proteins and Zip1-SNAP-IR700 for NIR-PIT. A major advantage of this approach is its modularity—only the Zip1-SNAP-IR700 component needs to be standardized, while the targeting scFv-Zip2 can be easily adapted for different cancer antigens. Coiled coil motifs, such as synthetic zipper domains, have been widely used in molecular engineering, including in chimeric antigen receptor (CAR) T cell therapy^42^ and enzymatic pathway engineering.^43^ Prior studies have demonstrated the successful application of synthetic coiled coil in these contexts.^35^^,^^44^^,^^45^

In this study, we developed three scFv-Zip2 fusion proteins targeting FOLR1, TROP2, and TF as well as Zip1-SNAP as therapeutic motif to conjugate IR700 site specifically, ensuring uniform labeling of each protein. Importantly, previous studies have demonstrated that SNAP-tag-mediated conjugation allows precise attachment of IR700 without adversely affecting target accessibility, biodistribution, phototoxic effects, or protein stability.^29^^,^^32^^,^^33^ While the ZIP1/ZIP2 coiled-coil pair demonstrated sufficient specificity and stability for use in our pre-targeting system, we acknowledge that quantitative binding affinity (Kd) for this pair has not been directly measured in the context of our SNAP-tag or scFv fusion constructs. Previous studies, such as Anderson et al.^46^ have shown that ZIP1/ZIP2 form parallel, dimeric coiled coils with strong binding affinity (dissociation constant, Kd, below 10 nM) and importantly, without self-association. Anderson et al. further confirmed the high specificity and affinity between Zip1-and Zip2-fused antibody fragments, reporting Kd values of 1.5 nM and 16 nM when Zip2- or Zip1-fused fragments were immobilized, respectively. The binding affinity may vary depending on the specific fusion constructs. Therefore, we plan to quantitatively determine the binding affinity of our pre-targeting complex using surface plasmon resonance and/or isothermal titration calorimetry in future studies. Moreover, studies need to perform by using non-complementary zipper control such as Zip1mut-SNAP-IR700.^46^ to further strengthen the evidence for the selectivity of the pre-targeting system. This study introduces the concept and provides preliminary *in vitro* validation; however, additional experiments to evaluate pharmacokinetic parameters such as half-life and biodistribution are necessary before advancing to further applications. Kobayashi and colleagues have shown that NIR-PIT induces more efficient photo-cytotoxicity than traditional photodynamic therapy, driven by photochemical damage that is independent of reactive oxygen species generation.^17^^,^^18^^,^^19^ Several studies have also reported the successful application of scFv molecules in targeted therapies.^29^^,^^32^^,^^33^^,^^34^

In this study, each pre-targeting complex exhibited specific cytotoxicity in ovarian cancer cell lines that is in line with our recent studies in breast cancer cells targeting TROP2.^36^ Among them, IGROV1 cells treated with the FOLR1-targeting complex (scFv-Farletuzumab-Zip2-Zip1-SNAP-IR700) displayed the highest toxicity, consistent with their FOLR1 expression level (IGROV1 ˃ OVCAR3 ˃ SKOV3). The A2780 cell line, which expresses low level of FOLR1, TROP2 and TF, showed no response to treatment, confirming the antigen-specific mechanism of cell death induced by our pre-targeting complex. IC_50_ values further supported these findings, with OVCAR3 and SKOV3 cells showing the highest sensitivity to TROP2 and TF-targeted complex (129.1 nM and 197.4 nM, respectively). OVCAR3 cells, which express moderate to high levels of all three antigens, responded to all three treatments, though slightly lower toxicity was observed compared to previous studies. This may be attributed to the additional binding step required in the pre-targeting system.^33^ Nevertheless, cell death was proportional to antigen expression and concentration of the pre-targeting complex. High expressing cells underwent extensive cell death, consistent with expression pattern and XTT assay results.

Interestingly, in SKOV3 cells, scFv-Sacituzumab-Zip2 exhibited stronger therapeutic effects, whereas scFv-Tisotumab-Zip2 showed weaker effects relative to their binding levels measured by flow cytometry. This suggests that therapeutic efficacy is influenced not only by surface antigen expression but also by factors such as internalization efficiency, subcellular trafficking, and accessibility of the NIR-PIT conjugates to their targets. For instance, scFv-Sacituzumab-Zip2 may internalize more efficiently or localize to compartments that enhance cytotoxicity, while scFv-Tisotumab-Zip2 may bind effectively yet internalize less or localize suboptimal. These considerations underscore that binding affinity alone may not fully predict NIR-PIT outcomes, and highlight the importance of assessing functional delivery and intracellular dynamics in evaluating therapeutic efficacy. In addition, while TF expression was detectable in both OVCAR3 and SKOV3 cell lines, the TF-targeting complex (scFv-Tisotumab-Zip2) demonstrated a lower IC_50_ in SKOV3 (403.8 nM) compared to OVCAR3 (642.9 nM). This may appear counterintuitive if based solely on TF expression levels; however, cytotoxic response in PIT is influenced by multiple factors beyond antigen abundance. These include differences in receptor accessibility, cell surface distribution, internalization rates, and the intrinsic susceptibility of each cell line to IR700-mediated photodamage. It is also possible that heterogeneity in TF expression within the OVCAR3 population contributes to a dampened average response. Such discrepancies have been previously noted in targeted therapies, where antigen density alone does not fully predict treatment efficacy.^47^ These findings highlight the importance of evaluating not only antigen expression levels but also cellular and biophysical context when interpreting IC_50_ values in pre-targeted PIT systems.

To validate the ICD, we measured the surface translocation of CRT, HSP70, and HSP90. In alignment with prior studies using mAb-IR700 and scFv-SNAP-IR700 conjugates,^48^^,^^49^ our pre-targeting system also induced significant upregulation of these DAMPs. Notably, SKOV3 cells treated with the TF-targeting complex showed a 4-fold, 10-fold, and 200-fold increase in CRT, HSP70, and HSP90 expression, respectively, compared to untreated controls. Lower DAMPs expression in IGROV1 cells may be attributed to morphological and phenotypic differences across cell lines. Although the advantages have been discussed, immunogenicity remains a concern for future clinical translation. Synthetic zippers may increase the risk of immune responses. Future *in vivo* studies need to be performed to evaluate the immunogenicity.

In conclusion, the synthetic zipper-mediated pre-targeting system described here represents a promising strategy for modular, antigen-specific NIR-PIT. Our *in vitro* results demonstrate its efficacy in selectively targeting and killing ovarian cancer cells, as well as inducing ICD. While these findings are encouraging, further *in vivo* studies need to assess the therapeutic potential and clinical applicability of this platform. The versatility of this system also allows its extension to other cancers by simply altering the scFv-Zip2 moiety—whether as an scFv, full IgG, or small peptide. Moreover, this pre-targeting strategy holds potential for broader applications, including radioimmunotherapy, antibody-drug conjugates, and diagnostic imaging *in vitro*.

### Limitations of the study

A limitation of the current study is that we did not assess HMGB-1 release, a key DAMP involved in ICD. Future studies should evaluate HMGB-1 to provide a more comprehensive understanding of NIR-PIT-induced ICD. In addition, functional validation of immune activation was not performed. Future studies are planned to include functional assays, such as dendritic cell co-culture to assess maturation markers and phagocytosis, as well as T cell activation assays. Another limitation of this study is that the assessment of pre-targeting system of NIR-PIT was confined to *in vitro* cell culture systems. Consequently, key *in vivo* factors such as pharmacokinetics, the impact of the tumor microenvironment, and potential off-target tissue toxicity, could not be fully evaluated. Future investigations utilizing xenograft or orthotopic cancer models will be necessary to assess biodistribution, therapeutic efficacy, and safety, thereby facilitating the translational advancement of these pre-targeting systems of NIR-PIT. A practical limitation of NIR-PIT is the requirement for effective delivery of NIR light to deep-seated tumors, such as those in the ovary. Solutions include the use of fiber optic diffusers, whole-body light systems, or minimally invasive delivery via endoscopes, catheters, or subcutaneous needles.^50^^,^^51^

## Resource availability

### Lead contact

Further information and requests for resources should be directed to and will be fulfilled by the lead contact, Ahmad Fawzi Hussain (ahmad.f.hussain@gyn.med.uni-giessen.de).

### Materials availability

This study did not generate new unique reagents.

### Data and code availability

• All data reported in this study are available from the lead contact upon request.
• This article does not report original code.
• Any additional information required to reanalyze the data reported in this article is available from the lead contact upon request.

## Acknowledgments

This work was funded in part by a research grant of the University Medical Center Giessen and Marburg (10.13039/501100009560UKGM) (8/2021 GI). We acknowledge the HPLC facility in 10.13039/100009091Justus Liebig University Giessen for performing all HPLC and mass spectrometry analysis.

## Author contributions

T.M.M.: investigation, writing – original draft preparation; C.Z.: investigation; W.S.: investigation; M.A.-R.: investigation and resources; N.E.-M.: visualization and writing – review & editing; F.Z.: visualization and writing – review & editing; I.M.-H.: conceptualization, supervision, and writing – review & editing; A.F.H.: conceptualization, funding acquisition, investigation, supervision, and writing – review & editing.

## Declaration of interests

T. M. Mohiuddin reports financial support was provided by research grant of the University Medical Center Giessen and Marburg (UKGM). The authors declare no competing interests.

## STAR★Methods

### Key resources table

**Table:** 

REAGENT or RESOURCE	SOURCE	IDENTIFIER
**Antibodies**		

FOLR1 mAb	Invitrogen	MA5-23917; RRID: AB_2609390
Trop2 (EGP-1) mAb	Invitrogen	14-6024-82; RRID: AB_10853488
CD142 Antibody	Miltenyi Biotec	130-098-741; RRID: AB_2655142
Goat anti-Mouse IgG (H+L) Highly Cross-Adsorbed Secondary Antibody, Alexa Fluor™ Plus 647	Invitrogen	A32728; RRID: AB_2633277
Anti-Hsp70-FITC	Miltenyi Biotec	130-105-548; RRID: AB_2652202
Anti-Hsp90-APC	Invitrogen	MA5-45102; RRID: AB_2931556
Human Calreticulin antibody	R&D	IC38981G; RRID: AB_3655647

**Experimental models: Cell lines**		

SKOV3	ATCC	HTB-77
OVCAR3	ATCC	HTB-161
IGROV1	Merck	SCC-203
A2780	ECACC	ECACC-93112519
HEK-293T	ATCC	CRL-11268

**Experimental models: Organisms/strains**		

One Shot™ MAX Efficiency™ DH5α-T1^R^ Competent Cells	Thermo	12297016
E. coli DH5α pMS-scFv-Farletuzumab-Zip2, Amp^R^	This paper	N/A
E. coli DH5α pMS-scFv-Sacituzumab-Zip2, Amp^R^	Zhang et al.	https://doi.org/10.1016/j.ejpb.2025.114794
E. coli DH5α pMS-scFv-Tisotumab-Zip2, Amp^R^	This paper	N/A
E. coli DH5α pMS-Zip1-SNAP, Amp^R^	Zhang et al.	https://doi.org/10.1016/j.ejpb.2025.114794
HEK293T-pMS-scFv-Farletuzumab-Zip2, Bleo^R^	This paper	N/A
HEK293T-pMS-scFv-Sacituzumab-Zip2, Bleo^R^	Zhang et al.	https://doi.org/10.1016/j.ejpb.2025.114794
HEK293T-pMS-scFv-Tisotumab-Zip2, Bleo^R^	This paper	N/A
HEK293T-pMS-Zip1-SNAP, Bleo^R^	Zhang et al.	https://doi.org/10.1016/j.ejpb.2025.114794

**Recombinant DNA**		

pMS-scFv-Farletuzumab-Zip2	This paper	N/A
pMS-scFv-Sacituzumab-Zip2	Zhang et al.	https://doi.org/10.1016/j.ejpb.2025.114794
pMS-scFv-Tisotumab-Zip2	This paper	N/A
pMS-Zip1-SNAP	Zhang et al.	https://doi.org/10.1016/j.ejpb.2025.114794

**Software and algorithms**		

FlowJo 10.7.1	BD	https://flowjo.com
GraphPad Prism 9.0.0	GraphPad	https://www.graphpad.com

**Other**		

IRdye700DX NHS	LI-COR	92970010
BG-PEG-NH2	NEB	S9150S

### Experimental model and study participant details

#### Cell lines and stains

Ovarian cancer cell lines SKOV3 (HTB-77), OVCAR3 (HTB-161), IGROV1 (SCC-203), A2780 (ECACC-93112519), and HEK293T (CRL-11268) were obtained from the American Type Culture Collection, the European Collection of Authenticated Cell Cultures, and Sigma-Aldrich. These cell lines were maintained in RPMI 1640 culture medium (Biowest) supplemented with 10% (v/v) fetal bovine serum (FBS) (Thermo Fisher Scientific) and 1% (v/v) penicillin-streptomycin (Thermo Fisher Scientific). Cultures were grown in a humidified incubator at 37 °C with 5% carbon dioxide and used for experiments within 30 passages. All cell lines were authenticated and tested negative for mycoplasma contamination (Eurofins Genomics, Germany) prior to use. One Shot™ MAX Efficiency™ DH5α-T1^R^ Competent Cells (Thermo Fisher Scientific, cat. no. 12297016) were used to clone the expression vector. Stains were cultured in LB media containing 100 μg/ml ampicillin overnight at 37 °C.

### Method details

#### Expression and enrichment of scFv-Zip2 and Zip1- SNAP

The Zip1-SNAP-tag and scFv-Zip2 fragments were inserted into the mammalian expression vector pMS. The plasmids were cloned in DH5α and isolated plasmids were transfected into HEK293T cells using Roti® Fect (Carl Roth, Karlsruhe, Germany). HEK293T cells expressing scFv-Zip2 and Zip1-SNAP were maintained in RPMI 1640 complete medium supplemented with 0.1% (v/v) zeocin (InvivoGen) to ensure selection of the transfected cells. The purification of C-terminal 6× His-tagged fusion proteins from cell-free supernatants was performed using an Äkta FPLC system (GE Healthcare Bio-Sciences AB, Uppsala, Sweden) and a Ni-NTA Superflow cartridge (Qiagen, Hilden, Germany). The enrichment of the fusion proteins was carried out using with different imidazole concentrations (10, 40 and 250 mM, respectively) and subsequent eluted fraction were collected during purification. After incubation with SNAP-Surface® Alexa Fluor® 488 (New England Biolabs, Ipswich, MA, USA) for 20 min at room temperature in the dark, the fractions were subjected to 10% SDS-PAGE to verify SNAP-tag activity and protein presence, followed by Coomassie Brilliant Blue staining.

#### Preparation of BG-IR700

IR700 (LI-COR Biosciences, Lincoln, NE, USA. #92970010) and BG-PEG-NHS (New England Biolabs, Ipswich, MA, USA, #S9150S) were conjugated by incubating two fold molar excess of BG-PEG-NHS in phosphate-buffered saline (PBS, pH 7.4) and allowed to react overnight at room temperature in the dark. The resulting BG-conjugated IR700 was then assessed and isolated using high-performance liquid chromatography (HPLC) on a Prontosil C-18 column (250 × 4.6 mm, 5 μm, 120 Å). Chromatographic separation was monitored at 286 nm and 680 nm to detect the BG linker, the native IR700 dye, and the BG-modified product. Purification of BG-PEG-IR700 was performed using a 55-minute gradient from buffer A (0.1 M triethylammonium acetate, TEAA) to buffer B (70% acetonitrile) at a flow rate of 1 mL/min. The mass of BG-IR700 was confirmed using a Bruker MicroTOF LC mass spectrometer with an electrospray ion source. All HPLC and mass spectrometry analysis were conducted by the HPLC facility, Institute of Organic Chemistry, Justus-Liebig-University.

#### Conjugation of scFv-Zip2 and Zip1-SNAP- fusion proteins with Alexa Flour488 NHS-Ester and BG-IR700

The scFv-Zip2 fusion proteins were labeled with Alexa Fluor™ 488 NHS-Ester (Succinimidylester) and Zip1-SNAP fusion proteins were conjugated with BG-IR700 by incubating at a 1:2 ratio for 2 h at room temperature in the dark. Unconjugated dyes were removed using 7K MWCO Zeba™ Spin Desalting Columns (Thermo Fisher Scientific). The labeled proteins were visualized after separation by SDS-PAGE and measured the concentration using bovine serum albumin standard.^37^

#### Expression level of target antigen and binding specificity of pre-targeting complex of NIR-PIT agents by flow cytometry

Cell surface expression of FOLR1, Trop2 and TF was analyzed by flow cytometry. SKOV3, OVCAR3, IGROV1 and A2780 cells (4 x 10^5^) were incubated with anti FOLR1 (FOLR1 mAb, clone 548908, 0.5 μg), anti-Trop2 (Trop2 mAb, clone MR54, 1 μg) and anti TF (CD142 mAb, clone HTF-1, 10 μL) (130-098-741) antibodies 200 μL of PBS for 30 min on ice. After washing twice with PBS, the cells were incubated with goat anti-mouse IgG (H+L) Highly Cross-Adsorbed Secondary Antibody conjugated with Alexa Fluor™ Plus 647 (0.25 μg, Invitrogen, #A32728) for 30 min on ice. After washing twice, cells were resuspended in 200 μL of PBS and analyzed by CytoFLEX S Flow Cytometers. The binding efficiency of the scFv-SNAP-IR700 agents was also determined by flow cytometry. SKOV3, OVCAR3, IGROV1 and A2780 were used to analyze the binding efficiency of 647 conjugated scFv-Farletuzumab-SNAP, scFv-Sacituzumab-SNAP and scFv-Tisotuzumab-SNAP. Cells (4 x 10^5^) were washed with 1 mL PBS twice, followed by incubation with 1 μg of scFv-SNAP-647 with PBS for 30 min on ice. Cells were washed with 1 mL PBS twice and resuspended in 200 μL of PBS and analyzed by CytoFLEX S Flow Cytometers. In addition, SKOV3, OVCAR3, IGROV1 and A2780 were used to measure the binding efficiency of pre-targeting complex after incubating with scFv-Farletuzumab-Zip2, scFv-Sacituzumab-Zip2 and scFv-Tisotuzumab-Zip2. Cells were washed and stained with 1 μg of Zip1-SNAP-IR700 with PBS for 30 min on ice. Cells were washed twice and resuspended and analyzed by CytoFLEX LX Flow Cytometers.

#### Binding and co-localization analysis by fluorescence microscopy

Ovarian cancer cells were seeded in black 96-well plate with a clear bottom to a density of 40,000 cells/well and incubated overnight at 37°C. Cells were washed with PBS twice and then incubated with 1 μg of each scFv-Farletuzumab-Zip2-488 or scFv-Sacituzumab-Zip2-488 or scFv-Tisotuzumab-Zip2-488on ice for 1 h. Then cells were washed with PBS twice, followed by incubating with Zip1-SNAP-IR700 for 1 h. After washing two times with PBS, cells were incubated with Hoechst 33342 nuclear staining (1 μg/mL, Thermo Fisher Scientific) for 10 min at room temperature. Cells were washed with PBS and visualized with a DMi8 S Live-cell microscope using a 100 × oil objective.

#### Cytotoxicity of pre-targeting complex of NIR-PIT by XTT assay

Ovarian cancer cells were seeded in 96-well plates at a density of 5000 cells/well and incubated at 37°C overnight. Cells were treated with 1600 nM of scFv-Zip2 for 3 h followed by incubation with serially diluted Zip1-SNAP fusion proteins, both with and without IR700 conjugation in 12.5, 25, 50, 100, 200, 400, 800, 1600 nM at 37°C for 1h. Cells treated with media or zeocin were used as negative and positive controls, respectively. Zeocin is an antibiotic commonly used for selection of stably transfected cells. To irradiate the cells, a LED with 670–710 nm wavelength range (L690-66-60; Marubeni America Co., New York, NY) and power density of 20 μW/cm^2^ at 400 mA CW (measured by a power meter PM 100; Thorlabs, Newton, NJ). Following NIR light exposure at a dose of 2 J/cm^2^, the cells were incubated in complete medium for 24 h. Cell viability was assessed by XTT kit (Cell Proliferation Kit II, Roche #11465015001) according to the manufacturer protocol. The data was analysed using GraphPad software.

#### Measurement of pre-targeting complex mediated cell death induction by annexin assay

Cell death induction mediated by pre-targeting complex was determined using SNAP-Surface® Alexa Fluor® 647 conjugated annexin V-SNAP fusion protein and propidium iodide described previously by Zhang et al. 2022.^37^ Cells were seeded in 24-well plates at a density of 50,000 cells/well in triplicates for overnight. Cells were washed and incubated with IR700 conjugated and unconjugated scFv-Zip2 fusion proteins in 1600 nM for 3 h. After light irradiation, cells were harvested and treated with 0.5 μg of annexin V-SNAP-647 for 30 min. Cells were washed and incubated with 0.1 μg of propidium iodide (Thermo Fisher Scientific) for 10 min and analyzed by CytoFLEX LX Flow Cytometers.

#### Measurement of DAMPs release by flow cytometry

SKOV3, OVCAR3 and IGROV1 cells were seeded in 24-well plates in triplicates for overnight. Cells were incubated with scFv-Tisotumab-Zip2, scFv-Sacituzumab-Zip2 and scFv-Farletuzumab-Zip2 in 1600 nM for 3 h followed by incubation with IR700-conjugated or unconjugated Zip1-SNAP. After 24 h NIR light irradiation, cells were harvested and incubated with 0.50 μg calreticulin (488-conjugated, Clone 681233, R&D Systems #IC38981G-100UG) antibody or 2 μL HSP70 (FITC-Conjugated, Miltenyi Biotech #130-105-548) antibody or 1 μg HSP90 (APC conjugated, H9010, Invitrogen #MA5-45102) antibody for 30 min. Cells were washed and resuspended and analyzed by CytoFLEX S Flow Cytometers. The number of the cells was expressed in percentages from the living gated cells and calreticulin, HSP70 and HSP90-specific MFI was calculated. The data was analyzed in FlowJo and GraphPad software.

### Quantification and statistical analysis

Data are presented as means with SD. Statistical analysis and drawing of graphs were performed with GraphPad software version 9.0.0 (GraphPad Software Inc., La Jolla, CA, USA). For multiple comparisons, a one-way ANOVA and Dunnett's multiple comparisons test was used. One tail nonparametric spearman correlation were used in correlation analysis. All of the statistical details of experiments are described in the figure legends including the statistical tests used, number of biological replicates. P values of less than 0.05 were considered significant.

## References

[1] Houghton J.L., Membreno R., Abdel-Atti D., Cunanan K.M., Carlin S., Scholz W.W., Zanzonico P.B., Lewis J.S., Zeglis B.M. (2017). Establishment of the In Vivo Efficacy of Pretargeted Radioimmunotherapy Utilizing Inverse Electron Demand Diels-Alder Click Chemistry. Mol. Cancer Ther..

[2] Cheal S.M., Chung S.K., Vaughn B.A., Cheung N.K.V., Larson S.M. (2022). Pretargeting: A Path Forward for Radioimmunotherapy. J. Nucl. Med..

[3] Shirasu N., Shibaguchi H., Yamada H., Kuroki M., Yasunaga S. (2019). Highly versatile cancer photoimmunotherapy using photosensitizer-conjugated avidin and biotin-conjugated targeting antibodies. Cancer Cell Int..

[4] Tienken L., Drude N., Schau I., Winz O.H., Temme A., Weinhold E., Mottaghy F.M., Morgenroth A. (2018). Evaluation of a Pretargeting Strategy for Molecular Imaging of the Prostate Stem Cell Antigen with a Single Chain Antibody. Sci. Rep..

[5] Rondon A., Schmitt S., Briat A., Ty N., Maigne L., Quintana M., Membreno R., Zeglis B.M., Navarro-Teulon I., Pouget J.P. (2019). Pretargeted radioimmunotherapy and SPECT imaging of peritoneal carcinomatosis using bioorthogonal click chemistry: probe selection and first proof-of-concept. Theranostics.

[6] Pimm M.V., Fells H.F., Perkins A.C., Baldwin R.W. (1988). Iodine-131 and indium-111 labelled avidin and streptavidin for pre-targetted immunoscintigraphy with biotinylated anti-tumour monoclonal antibody. Nucl. Med. Commun..

[7] Hapuarachchige S., Si G., Huang C.T., Lesniak W.G., Mease R.C., Guo X., Gabrielson K., Artemov D. (2021). Dual-Modality PET-SPECT Image-Guided Pretargeting Delivery in HER2(+) Breast Cancer Models. Biomacromolecules.

[8] Wei R., Dong Y., Tu Y., Luo S., Pang X., Zhang W., Yao W., Tang W., Yang H., Wei X. (2021). Bioorthogonal Pretargeting Strategy for Anchoring Activatable Photosensitizers on Plasma Membranes for Effective Photodynamic Therapy. ACS Appl. Mater. Interfaces.

[9] Bailly C., Bodet-Milin C., Rousseau C., Faivre-Chauvet A., Kraeber-Bodéré F., Barbet J. (2017). Pretargeting for imaging and therapy in oncological nuclear medicine. EJNMMI Radiopharm. Chem..

[10] Pagel J.M., Orgun N., Hamlin D.K., Wilbur D.S., Gooley T.A., Gopal A.K., Park S.I., Green D.J., Lin Y., Press O.W. (2009). A comparative analysis of conventional and pretargeted radioimmunotherapy of B-cell lymphomas by targeting CD20, CD22, and HLA-DR singly and in combinations. Blood.

[11] Verhoeven M., Seimbille Y., Dalm S.U. (2019). Therapeutic Applications of Pretargeting. Pharmaceutics.

[12] Hapuarachchige S., Huang C.T., Donnelly M.C., Bařinka C., Lupold S.E., Pomper M.G., Artemov D. (2020). Cellular Delivery of Bioorthogonal Pretargeting Therapeutics in PSMA-Positive Prostate Cancer. Mol. Pharm..

[13] Karacay H., Sharkey R.M., McBride W.J., Griffiths G.L., Qu Z., Chang K., Hansen H.J., Goldenberg D.M. (2002). Pretargeting for cancer radioimmunotherapy with bispecific antibodies: role of the bispecific antibody's valency for the tumor target antigen. Bioconjug. Chem..

[14] Weiden P.L., Breitz H.B. (2001). Pretargeted radioimmunotherapy (PRIT) for treatment of non-Hodgkin's lymphoma (NHL). Crit. Rev. Oncol. Hematol..

[15] Westerlund K., Altai M., Mitran B., Konijnenberg M., Oroujeni M., Atterby C., de Jong M., Orlova A., Mattsson J., Micke P. (2018). Radionuclide Therapy of HER2-Expressing Human Xenografts Using Affibody-Based Peptide Nucleic Acid-Mediated Pretargeting: In Vivo Proof of Principle. J. Nucl. Med..

[16] Xu X., Zhang H., Cao Y., Liu W., Chen Z., Li C. (2025). Cell Membrane-Targeted J-Aggregation Strategy for Synergistic Immune Checkpoint Degradation and Immunogenic Pyroptosis to Augment Tumor Immunotherapy. Angew. Chem. Int. Ed. Engl..

[17] Mitsunaga M., Ogawa M., Kosaka N., Rosenblum L.T., Choyke P.L., Kobayashi H. (2011). Cancer cell-selective *in vivo* near infrared photoimmunotherapy targeting specific membrane molecules. Nat. Med..

[18] Kobayashi H., Choyke P.L. (2019). Near-Infrared Photoimmunotherapy of Cancer. Acc. Chem. Res..

[19] Kobayashi H., Furusawa A., Rosenberg A., Choyke P.L. (2021). Near-infrared photoimmunotherapy of cancer: a new approach that kills cancer cells and enhances anti-cancer host immunity. Int. Immunol..

[20] Mohiuddin T.M., Zhang C., Sheng W., Al-Rawe M., Zeppernick F., Meinhold-Heerlein I., Hussain A.F. (2023). Near Infrared Photoimmunotherapy: A Review of Recent Progress and Their Target Molecules for Cancer Therapy. Int. J. Mol. Sci..

[21] Cheung A., Bax H.J., Josephs D.H., Ilieva K.M., Pellizzari G., Opzoomer J., Bloomfield J., Fittall M., Grigoriadis A., Figini M. (2016). Targeting folate receptor alpha for cancer treatment. Oncotarget.

[22] Goldenberg D.M., Stein R., Sharkey R.M. (2018). The emergence of trophoblast cell-surface antigen 2 (TROP-2) as a novel cancer target. Oncotarget.

[23] Wen Y., Ouyang D., Zou Q., Chen Q., Luo N., He H., Anwar M., Yi W. (2022). A literature review of the promising future of *TROP2*: a potential drug therapy target. Ann. Transl. Med..

[24] Wu B., Yu C., Zhou B., Huang T., Gao L., Liu T., Yang X. (2017). Overexpression of TROP2 promotes proliferation and invasion of ovarian cancer cells. Exp. Ther. Med..

[25] Xu N., Zhang Z., Zhu J., Xu L., Li Y., Duan L., Mao Y., Li H. (2016). Overexpression of trophoblast cell surface antigen 2 as an independent marker for a poor prognosis and as a potential therapeutic target in epithelial ovarian carcinoma. Int. J. Exp. Pathol..

[26] Hisada Y., Mackman N. (2019). Tissue Factor and Cancer: Regulation, Tumor Growth, and Metastasis. Semin. Thromb. Hemost..

[27] Cocco E., Varughese J., Buza N., Bellone S., Lin K.Y., Bellone M., Todeschini P., Silasi D.A., Azodi M., Schwartz P.E. (2011). Tissue factor expression in ovarian cancer: implications for immunotherapy with hI-con1, a factor VII-IgGF(c) chimeric protein targeting tissue factor. Clin. Exp. Metastasis.

[28] Sato K., Watanabe R., Hanaoka H., Harada T., Nakajima T., Kim I., Paik C.H., Choyke P.L., Kobayashi H. (2014). Photoimmunotherapy: comparative effectiveness of two monoclonal antibodies targeting the epidermal growth factor receptor. Mol. Oncol..

[29] Hussain A.F., Heppenstall P.A., Kampmeier F., Meinhold-Heerlein I., Barth S. (2019). One-step site-specific antibody fragment auto-conjugation using SNAP-tag technology. Nat. Protoc..

[30] Sheng W., Zhang C., Mohiuddin T.M., Al-Rawe M., Schmitz R., Niebert M., Konrad L., Wagner S., Zeppernick F., Meinhold-Heerlein I., Hussain A.F. (2025). Development of SNAP-Tag Based Nanobodies as Secondary Antibody Mimics for Indirect Immunofluorescence Assays. Cells.

[31] Mohiuddin T.M., Sheng W., Zhang C., Al-Rawe M., Tchaikovski S., Zeppernick F., Meinhold-Heerlein I., Hussain A.F. (2025). Multiplex Immunofluorescence Reveals Therapeutic Targets EGFR, EpCAM, Tissue Factor, and TROP2 in Triple-Negative Breast Cancer. Int. J. Mol. Sci..

[32] Amoury M., Bauerschlag D., Zeppernick F., von Felbert V., Berges N., Di Fiore S., Mintert I., Bleilevens A., Maass N., Bräutigam K. (2016). Photoimmunotheranostic agents for triple-negative breast cancer diagnosis and therapy that can be activated on demand. Oncotarget.

[33] Bauerschlag D., Meinhold-Heerlein I., Maass N., Bleilevens A., Bräutigam K., Al Rawashdeh W., Di Fiore S., Haugg A.M., Gremse F., Steitz J. (2017). Detection and Specific Elimination of EGFR^+^ Ovarian Cancer Cells Using a Near Infrared Photoimmunotheranostic Approach. Pharm. Res..

[34] Burley T.A., Mączyńska J., Shah A., Szopa W., Harrington K.J., Boult J.K.R., Mrozek-Wilczkiewicz A., Vinci M., Bamber J.C., Kaspera W., Kramer-Marek G. (2018). Near-infrared photoimmunotherapy targeting EGFR-Shedding new light on glioblastoma treatment. Int. J. Cancer.

[35] Reinke A.W., Grant R.A., Keating A.E. (2010). A synthetic coiled-coil interactome provides heterospecific modules for molecular engineering. J. Am. Chem. Soc..

[36] Zhang C., Sheng W., Mohiuddin T.M., Al-Rawe M., Schmitz R., Niebert M., Zeppernick F., Meinhold-Heerlein I., Hussain A.F. (2025). A coiled coil-based pre-targeting drug delivery system for precise treatment of breast cancer. Eur. J. Pharm. Biopharm..

[37] Zhang C., Sheng W., Al-Rawe M., Mohiuddin T.M., Niebert M., Zeppernick F., Meihold-Heerlein I., Hussain A.F. (2022). EpCAM- and EGFR-Specific Antibody Drug Conjugates for Triple-Negative Breast Cancer Treatment. Int. J. Mol. Sci..

[38] Heemskerk B., Kvistborg P., Schumacher T.N.M. (2013). The cancer antigenome. EMBO J..

[39] Schoffelen R., Woliner-van der Weg W., Visser E.P., Goldenberg D.M., Sharkey R.M., McBride W.J., Chang C.H., Rossi E.A., van der Graaf W.T.A., Oyen W.J.G., Boerman O.C. (2014). Predictive patient-specific dosimetry and individualized dosing of pretargeted radioimmunotherapy in patients with advanced colorectal cancer. Eur. J. Nucl. Med. Mol. Imaging.

[40] Touchefeu Y., Bailly C., Frampas E., Eugène T., Rousseau C., Bourgeois M., Bossard C., Faivre-Chauvet A., Rauscher A., Masson D. (2021). Promising clinical performance of pretargeted immuno-PET with anti-CEA bispecific antibody and gallium-68-labelled IMP-288 peptide for imaging colorectal cancer metastases: a pilot study. Eur. J. Nucl. Med. Mol. Imaging.

[41] Kraeber-Bodéré F., Rousseau C., Bodet-Milin C., Frampas E., Faivre-Chauvet A., Rauscher A., Sharkey R.M., Goldenberg D.M., Chatal J.F., Barbet J. (2015). A pretargeting system for tumor PET imaging and radioimmunotherapy. Front. Pharmacol..

[42] Cho J.H., Collins J.J., Wong W.W. (2018). Universal Chimeric Antigen Receptors for Multiplexed and Logical Control of T Cell Responses. Cell.

[43] Bozhueyuek K.A.J., Watzel J., Abbood N., Bode H.B. (2021). Synthetic Zippers as an Enabling Tool for Engineering of Non-Ribosomal Peptide Synthetases∗. Angew. Chem. Int. Ed. Engl..

[44] Grigoryan G., Reinke A.W., Keating A.E. (2009). Design of protein-interaction specificity gives selective bZIP-binding peptides. Nature.

[45] Thompson K.E., Bashor C.J., Lim W.A., Keating A.E. (2012). SYNZIP protein interaction toolbox: *in vitro* and *in vivo* specifications of heterospecific coiled-coil interaction domains. ACS Synth. Biol..

[46] Anderson G.P., Shriver-Lake L.C., Liu J.L., Goldman E.R. (2018). Orthogonal Synthetic Zippers as Protein Scaffolds. ACS Omega.

[47] Chari R.V.J., Miller M.L., Widdison W.C. (2014). Antibody-drug conjugates: an emerging concept in cancer therapy. Angew. Chem. Int. Ed. Engl..

[48] Ogawa M., Tomita Y., Nakamura Y., Lee M.J., Lee S., Tomita S., Nagaya T., Sato K., Yamauchi T., Iwai H. (2017). Immunogenic cancer cell death selectively induced by near infrared photoimmunotherapy initiates host tumor immunity. Oncotarget.

[49] Mohiuddin T.M., Zhang C., Sheng W., Al-Rawe M., Schmitz R., Niebert M., El-Merhie N., Zeppernick F., Meinhold-Heerlein I., Hussain A.F. (2025). Near-infrared photoimmunotherapy for effective elimination of ovarian cancer cells by inducing immunogenic cell death. Mol. Ther. Oncol..

[50] Tsukamoto T., Fujita Y., Shimogami M., Kaneda K., Seto T., Mizukami K., Takei M., Isobe Y., Yasui H., Sato K. (2022). Inside-the-body light delivery system using endovascular therapy-based light illumination technology. EBioMedicine.

[51] Nagaya T., Okuyama S., Ogata F., Maruoka Y., Choyke P.L., Kobayashi H. (2018). Endoscopic near infrared photoimmunotherapy using a fiber optic diffuser for peritoneal dissemination of gastric cancer. Cancer Sci..

