# Supplementary material for: Synthetic zipper mediated pre-targeting system for near-infrared photoimmunotherapy
Source: iScience. 2025 Dec 29;29(2):114558. doi: 10.1016/j.isci.2025.114558 (PMC12830222; doi:10.1016/j.isci.2025.114558)
Supplement: Document S1. Figures S1–S4 and Data S1 [file mmc1.pdf]

iScience, Volume *29*

Supplemental information

Synthetic zipper mediated pre-targeting system for near-infrared photoimmunotherapy

T.M. Mohiuddin, Chaoyu Zhang, Wenjie Sheng, Marwah Al-Rawe, Natalia El-Merhie, Felix Zeppernick, Ivo Meinhold-Heerlein, and Ahmad Fawzi Hussain

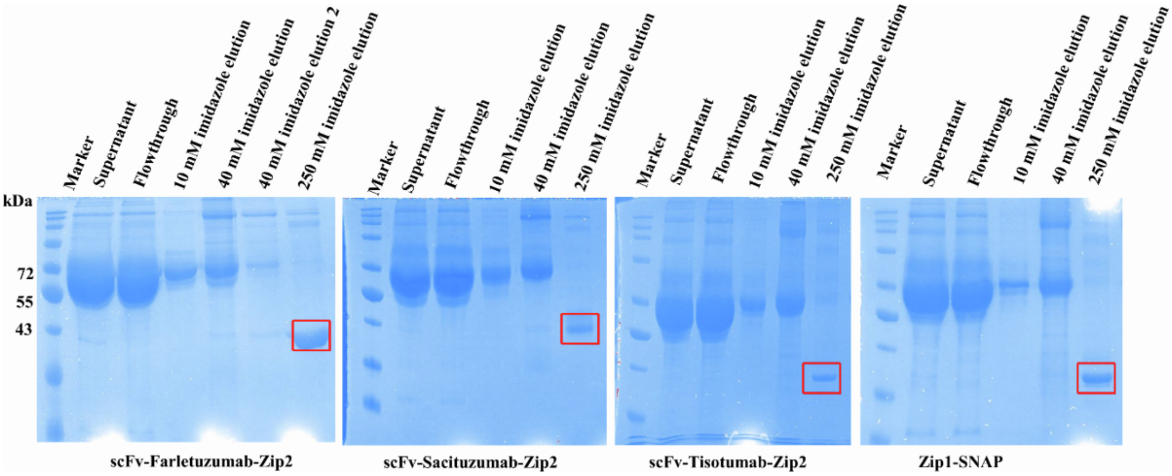

Figure S1. Enrichment of scFv-Farletuzumab-Zip2, scFv-Sacituzumab-Zip2, scFv-Tisotumab-Zip2 and Zip1-SNAP by nickel NTA using His-tagged and confirmed by SDS-PAGE by Coomassie blue staining and visualized with ChemiDoc XRS+ System. Protein standard broad range (11-250 kDa) were used as marker. The red box indicates the corresponding protein bands.

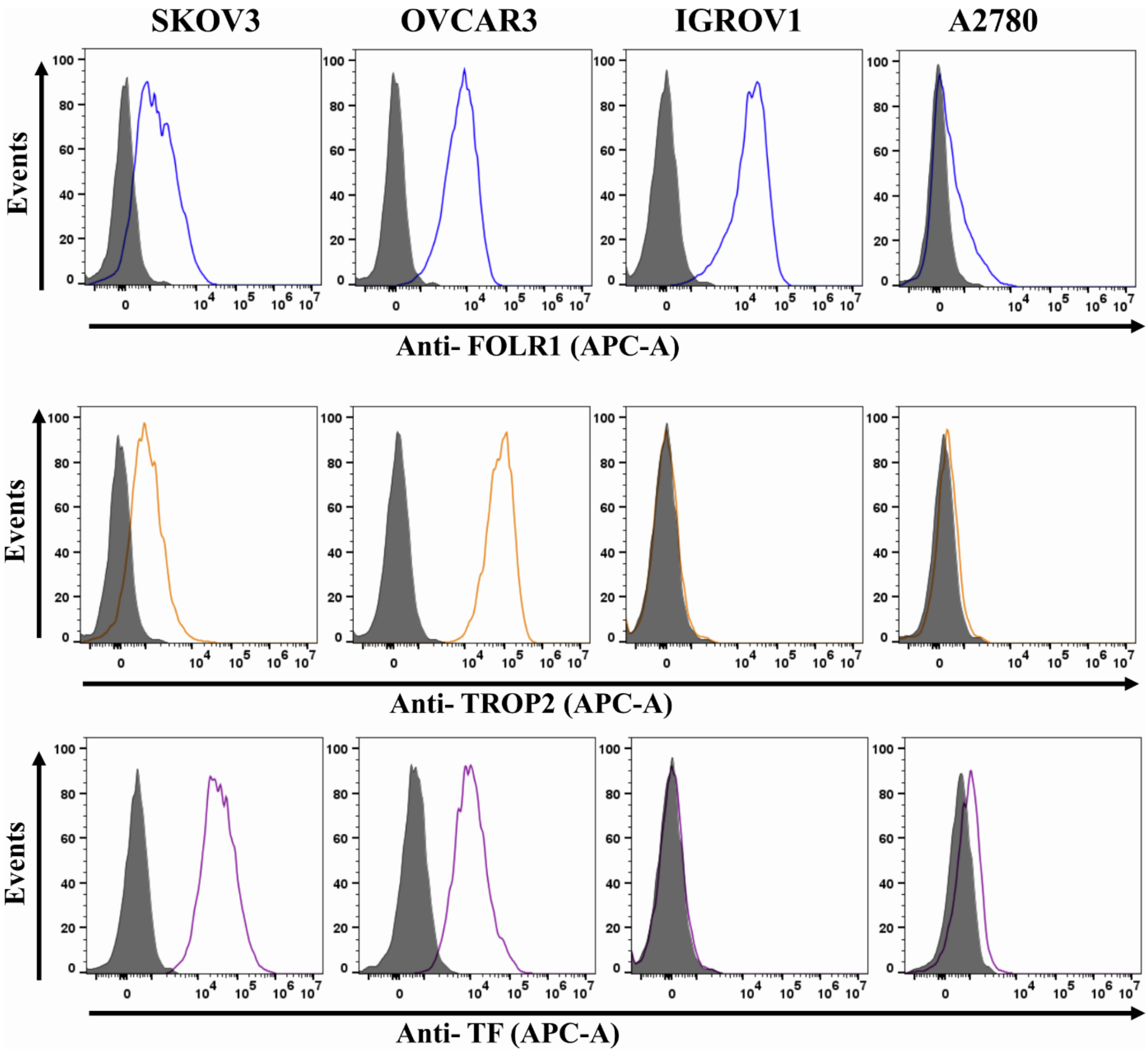

Figure S2. Expression patterns of FOLR1, TROP2 and TF in ovarian cancer cell lines. The flow cytometric histogram represents the expression of FOLR1, TROP2 and TF in ovarian cancer cells. Filled gray curves represent untreated cells and blue, brown and magenta curves represent cells incubated with anti FOLR1, anti-TROP2 antibodies and anti TF antibodies, respectively followed by incubation with the secondary antibody (Goat anti-Mouse IgG Highly Cross-Adsorbed Secondary Antibody, Alexa Fluor™ Plus 647).

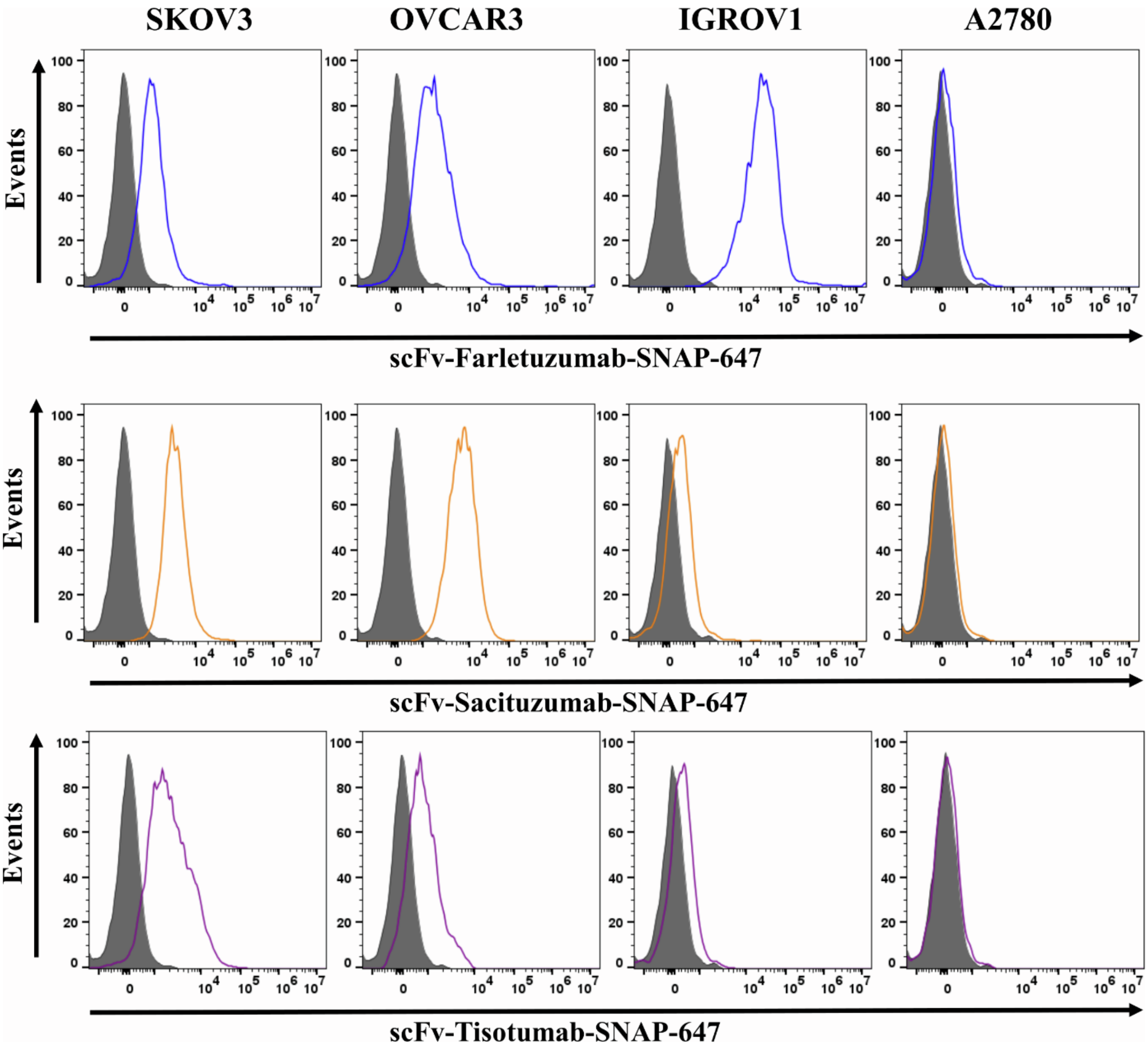

Figure S3. Specific binding of 647 conjugated scFv-SNAP tag to ovarian cancer cells. The flow cytometric histogram represents the binding of scFv-SNAP-647 to ovarian cancer cells. Filled gray curves represent untreated cells and blue, brown and magenta curves represent cells incubated with 0.5 µg/mL of the scFv-Farletuzumab-SNAP-647, scFv-Sacituzumab-SNAP-647 and scFv-Tisotumab-SNAP-647, respectively.

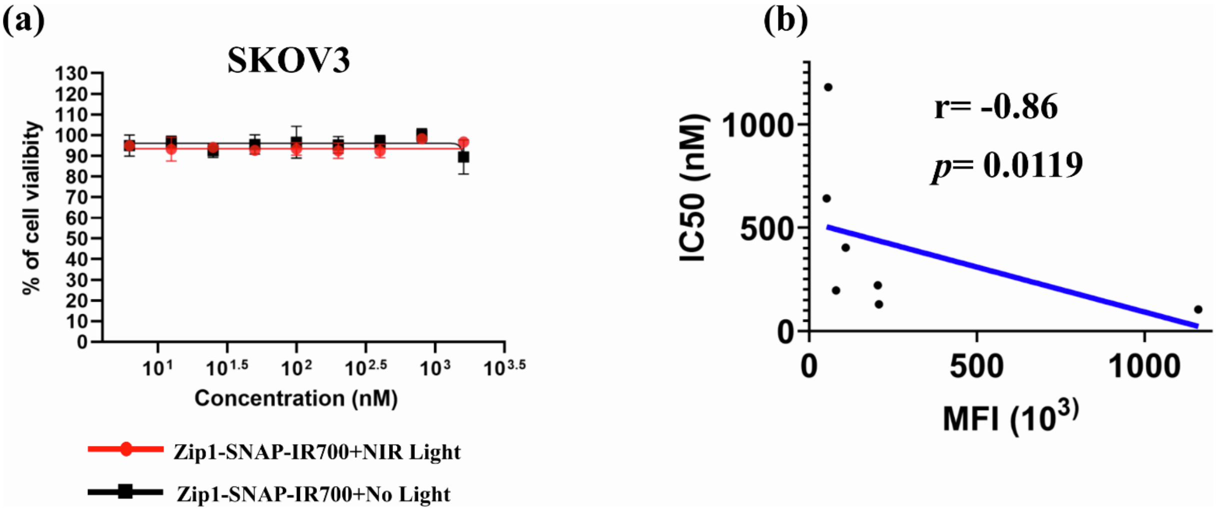

Figure S4. Cytotoxicity induced by Zip1-SNAP-IR700 and correlation of IC_{50} with target antigen expression. (a) SKOV3 cells were also treated with increasing concentration of (12.5, 25, 50, 100, 200, 400, 800, 1600 nM) Zip1-SNAP-IR700. Cells were then exposed or not exposed to NIR light (690–710 nm LED, 2 J/cm^2) and incubated at 37°C for 24 h. Cell viability was assessed using the XTT Cell Proliferation Kit II. Data represent mean ± SD. (b) Correlation of IC_{50} with MFI of target antigen. IC_{50} of <1600 were not included in this analysis. Spearman correlation plots illustrate the association of IC_{50} with target antigen expression. The blue line indicates the fitted linear regression line between IC_{50} with target antigen expression.

Data S1: Amino acid sequence of scFv-Farletuzumab, scFv-Sacituzumab, scFv-Tisotumab, Zip2, Zip1 and SNAPf.

scFv-Farletuzumab

EVQLVESGGGVVQPGRSLRLSCSASGFTFSGYGLSWVRQAPGKGLEWVAMISSGGSYTYYA
DSVKGRFAISRDNAKNTLFLQMDSLRPEDTGVYFCARHGDDPAWFAYWGQGTPVTVSSGGG
GSGGGGSGGGGSDIQLTQSPSSLSASVGDRVTITCSVSSSISSNNLHWYQQKPGKAPKPWIYGT
SNLASGVPSRFSGSGSGTDYTFTISSLQPEDIATYYCQQWSSYPYMYTFGQGTKVEIK

scFv-Sacituzumab

QVQLQQSGSELKKPGASVKVSCKASGYTFTNYGMNWVKQAPGQGLKWMGWINTYTGEPT
YTDDFKGRFAFSLDTSVSTAYLQISSLKADDTAVYFCARGGFGSSYWYFDVWGQGSLVTVSS
GGGGSGGGGSGGGGSDIQLTQSPSSLSASVGDRVSITCKASQDVSIAVAWYQQKPGKAPKLLI
YSASYRYTGVPDRFSGSGSGTDFTLTISSLQPEDFAVYYCQQHYITPLTFGAGTKVEIK

scFv-Tisotumab

EVQLLESGGGLVQPGGSLRLSCAASGFTFSNYAMSWVRQAPGKGLEWVSSISGSGDYTYYTD
SVKGRFTISRDNSKNTLYLQMNSLRAEDTAVYYCARSPWGYYLDSWGQGTLVTVSSGGGGS
GGGGSGGGGSDIQMTQSPPSLSASAGDRVTITCRASQGISSRLAWYQQKPEKAPKSLIYAASS
LQSGVPSRFSGSGSGTDFTLTISSLQPEDFATYYCQQYNSYPYTFGQGTKLEIK

Zip2

ARNAYLRKKIARLKKDNLQLERDEQNLEKIIANLRDEIARLENEVASHEQ

Zip1

NLVAQLENEVASLENENETLKKKNLHKKDLIAYLEKEIANLRKKIEE

SNAPf

MDKDCEMKRTTLDSPLGKLELSGCEQGLHEIKLLGKGTSAADAVEVPAPAAVLGGPEPLMQ
ATAWLNAYFHQPEAIEEFPVPALHHPVFQQESFTRQVLWKLLKVVKFGEVISYQQLAALAGN
PAATAAVKTALSGNPVPILIPCHRVVSSSGAVGGYEGGLAVKEWLLAHEGHRLGKPGL
